# The B-Type Cyclin CYCB1-1 Regulates Embryonic Development and Seed Size in Maize

**DOI:** 10.3390/ijms23115907

**Published:** 2022-05-25

**Authors:** Bingbing Zhao, Miaoyi Zhou, Wen Ren, Hanshuai Li, Qian Zhang, Guangming He, Ya Liu, Haohua He

**Affiliations:** 1 Key Laboratory of Crop Physiology, Ecology and Genetic Breeding Ministry of Education, College of Agronomy, Jiangxi Agricultural University, Nanchang 330046, China; bing3015@126.com; 2 Beijing Key Laboratory of Maize DNA Fingerprinting and Molecular Breeding, Maize Research Institute, Beijing Academy of Agriculture & Forestry Sciences, Beijing 100097, China; monazhou001@126.com (M.Z.); renwen@maizedna.org (W.R.); lhswgy@126.com (H.L.); zg0502q@163.com (Q.Z.); 3 College of Life Science, Yangtze University, Jingzhou 434025, China; 4 Peking-Tsinghua Center for Life Sciences, School of Life Sciences and School of Advanced Agriculture Sciences, Peking University, Beijing 100871, China; heguangming@pku.edu.cn

**Keywords:** cell cycle, B-type cyclin, embryo development, seed size, maize

## Abstract

Progress through the cell cycle is a critical process during plant embryo and seed development and its progression is regulated by cyclins. Despite extensive study of cyclins in other systems, their role in embryo and seed development of maize is unclear. In this study, we demonstrate that *ZmCYCB1-1* overexpression significantly accelerated embryo growth and increased seed size. In situ hybridization and toluidine blue staining indicated that *ZmCYCB1-1* was highly expressed in the plumule of embryos, and the cells of the plumule were smaller, denser, and more regularly arranged in *ZmCYCB1-1* overexpression plants. Overexpression of *ZmCYCB1-1* in maize also resulted in an increased ear length and enhanced kernel weight by increasing kernel width. Transcriptome analysis indicated that the overexpression of *ZmCYCB1-1* affected several different metabolic pathways, including photosynthesis in embryos and leaves, and lipid metabolism in leaves. Conversely, knocking out *ZmCYCB1-1* resulted in plants with slow growth. Our results suggest that *ZmCYCB1-1* regulates embryo growth and seed size, making it an ideal target for efforts aimed at maize yield improvement.

## 1. Introduction

The cell cycle plays a critical role in regulating the activities of plants, including seed germination, growth, and organ formation [1]. This cycle consists of four phases: gap1 phase (G1), DNA synthesis phase (S), gap2 phase (G2), and mitosis phase (M) [2]. Like other eukaryotes, the plant core cell cycle machinery is composed of cyclins (CYCs) and their associated cyclin-dependent kinases (CDKs) [3]. CYCs and CDKs combine to form specific CYC-CDK complexes at different cell cycle stages, regulating the transition between G1/S and G2/M phase, and controlling cell cycle progression as well as cell proliferation and differentiation [4,5].

Cyclin was first discovered in sea urchins [6], and the first plant cyclins were isolated in soybeans and carrots [6]. Since their initial discovery, cyclins have been studied extensively in model systems such as Arabidopsis [7,8,9,10]. Cyclin proteins typically contain a conserved N-terminal domain of approximately 100 amino acids, which may be related to their ability to bind to and regulate CDK function [8].

Several plant cyclin genes have been identified through systematic comparisons to known eukaryotic cyclins. In Arabidopsis, 49 cyclins have been identified, they are divided into ten subtypes: A, B, C, D, H, L, T, U, SDS, and J18. Among them, CYCAs and CYCBs can be further subdivided into three categories, CYCA1-A3 and CYCB1-B3. CYCDs are divided into seven categories including CYCD1-D7 [4,8]. In rice, 49 cyclins have been identified, and they are divided into nine subtypes: A, B, D, F, H, L, P, T, and SDS. Rice CYCAs can be further subdivided into CYCA1-A3, whereas CYCBs include CYCB1 and CYCB2, and CYCDs are divided into CYCD1-D7 [11]. A total of 59 cyclins have been identified in maize, including six subtypes: A, B, D, F, SDS, and T. Maize CYCAs can be further subdivided into CYCA1-A3, whereas CYCBs consist of CYCB1 and CYCB2, and CYCDs are divided into CYCD1-D7 [12].

Different cyclins exhibit distinct expression patterns during cell cycle progression. Cyclin A is expressed in late G1 and throughout S phases [13,14], whereas Cyclin B is expressed specifically in late G2- and M-phases during the cell cycle [15]. Cyclin D is involved in cell cycle regulation during the G1/S transition [2]. Experiments using synchronized tobacco cell suspensions revealed that *NtCYCB1-1* was not expressed in G1 and S phases but was observed in late G2 and early M phases, before disappearing in the G1 phase of a new cell cycle. Furthermore, injection of *NtCYCB1-1* into frog oocytes enabled cells to overcome the G2/M checkpoint [16]. Ectopic expression of the *CYCB1-2* gene in trigeminal hair cells that normally undergo nuclear re-replication has been shown to shift the nuclear re-replication cycle to the division cycle and produce multicellular trigeminal hairs, indicating that plant CYCB1 is involved in the regulation of G2/M phase [17]. Studies using synchronized cultured suspension cells in several plant species have shown that CYCB is not expressed prior to the S phase but begins to be transcribed in the G2 phase and slowly increases to its peak level in the early M phase. As the M phase progresses, the expression of CYCB then gradually decreases and finally disappears [16,18,19,20].

Due to the importance of cell cycle progression in many facets of plant growth and development, a better understanding of cyclins is critical. In angiosperms, seed development begins with double fertilization, and seed development then proceeds based on cell cycle progression. *OsCYCB1-1* has been shown to regulate embryo and endosperm development. In rice, RNAi inhibition of the *CYCB1-1* gene results in abnormal endosperm development within 3 days after pollination (DAP) but does not affect the embryo at this time point. At 9 DAP, the endosperm of seeds with *OsCYCB1-1* inhibition almost disappeared, while the size of the embryos increased 3–4 fold compared with control plants [21,22]. A genome-wide association study (GWAS) of seed size in 191 Arabidopsis accessions detected a significant association with the *CYCB1-4* locus, and natural variations in *CYCB1-4* have been shown to significantly influence seed size [23]. Transgenic overexpression of *CYCB1-4* altered normal development, increased seed size, and resulted in higher yields. By contrast, *cycb1-4* mutants have smaller seeds, with the effect being especially pronounced in a large-seed accession.

As an important food and feed crop throughout the world, maize (*Zea mays* L.) is one of the most significant sources of carbohydrates for humans and livestock [24]. Seed size selection is an extremely important aspect of maize breeding, affecting both kernel yield and quality. Recent studies have shown that B-type cyclins regulate kernel size in *Arabidopsis thaliana* [23], but their roles in maize are still poorly understood. In this study, we investigated a B-type cyclin, *CYCB1-1*, to better understand its functions and regulatory mechanisms. We generated overexpression and knockout plants and characterized changes in these aspects in plant growth, embryo regeneration, and seed size. Taken together, our results represent a significant step toward better understanding the role of cyclins in maize development.

## 2. Results

### 2.1. Cluster Analysis of B-Type Cyclins in Maize, Rice, and Arabidopsis

**Figure 1:**
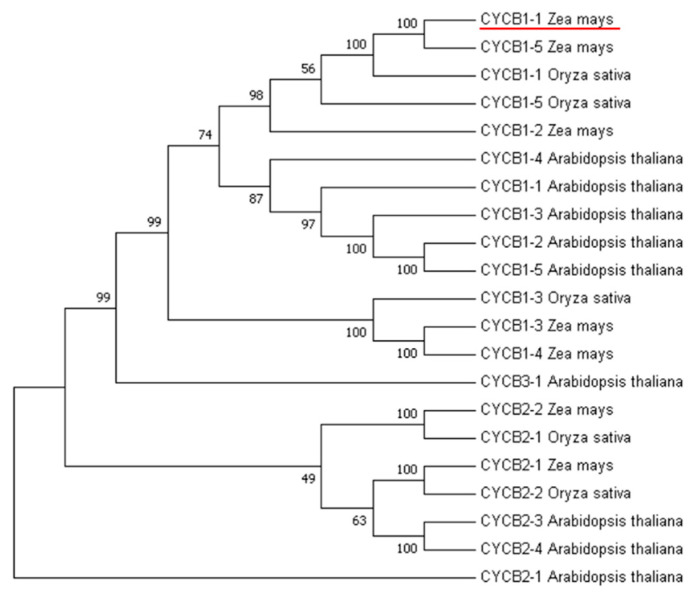
Phylogenetic analysis of CYCB gene families in maize, rice, and Arabidopsis.

For B-type cyclin cluster analysis, we used nine genes in Arabidopsis, five genes in rice, and seven genes in maize. This analysis revealed that the *CYCB1-1* gene in maize had the highest homology with the *CYCB1-1* gene in rice (Figure 1). The *CYCB1-1* gene of rice is distributed on chromosomes 1 and encodes a protein with length of 448 amino acid residues, a molecular weight of 49.5 kDa and a PI value of 9.12. The *CYCB1-1* gene of maize is distributed on chromosomes 8 and encodes a 50 kDa protein comprised of 449 amino acid residues and PI value of 9.19 (Table 1).

### 2.2. Generation of ZmCYCB1-1 Overexpression Plants

**Figure 2:**
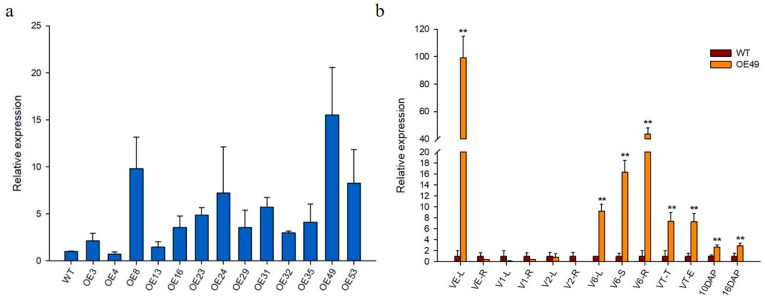
(**a**) Relative expression of *ZmCYCB1-1* in the 13 transgenic lines and wild type (WT). (**b**) Relative expression levels of *ZmCYCB1-1* normalized to WT in different growth stages in OE49. L: leaves; R: roots; S: stems; T: tassel; E: ears. Students *t* test, ** *p* < 0.01. The expression level of *ZmCYCB1-1* in WT in (**a**,**b**) set to 1.

The 2323 bp full-length genomic sequence of *ZmCYCB1-1*, which contains nine exons and eight introns, was obtained from the maize inbred line ZPM1. The pUbi::ZmCYCB1-1 vector was delivered into KN5585 via Agrobacterium-mediated transformation, and 13 independent transgenic lines with a single copy of the transgene were obtained. Relative gene expression levels in the leaves of wild-type (WT) and transgenic lines were analyzed by qRT-PCR analyses, which indicated that *ZmCYCB1-1* had varying levels of expression in all transgenic lines. OE49 had the highest expression levels (15-fold higher than WT), whereas the ZmCYCB1-1 transcript level in OE8, OE53, and OE24 was 9.8-, 8.2-, and 7.2-fold higher than that in WT, respectively (Figure 2a).

To investigate the tissue-specific expression profile of *ZmCYCB1-1*, tissues from WT and OE49 plants at different stages of growth were used for qRT-PCR analysis (Figure 2b). *ZmCYCB1-1* had the highest expression level at the vegetative emergence (VE) stage in OE49 leaves compared with WT leaves (99-fold of the WT). At the V1 and V2 stages, the leaves and roots of WT and OE49 showed no significant difference in *ZmCYCB1-1* expression. When the plants were at the jointing stage, *ZmCYCB1-1* expression increased significantly in the leaves, stems, and roots of OE49 (9-fold, 16-fold, and 43-fold higher than the WT, respectively). The expression level of *ZmCYCB1-1* in OE49 plants was 7-fold higher than that in the control plants in both tassel and female ears at the tasseling (VT) stage. A 3-fold increase in *ZmCYCB1-1* expression was observed in seeds at 10 DAP and 16 DAP.

### 2.3. Overexpression of ZmCYCB1-1 Accelerates the Growth of Isolated Embryos

**Figure 3:**
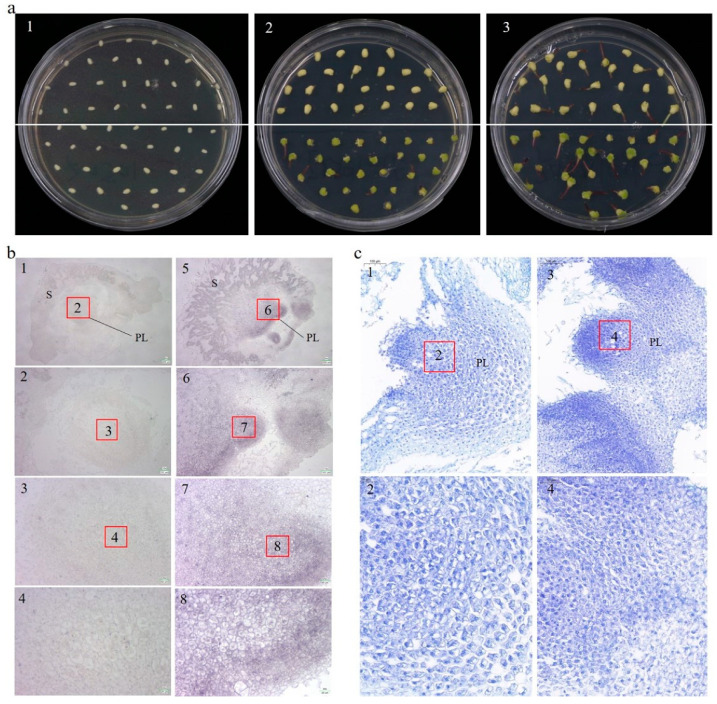
(**a**) Germination of OE49 and WT embryos on MS medium. (**a1**–**a3**). Germination of embryos after 0 days, 7 days and 10 days. WT and OE49 embryos are above and below the white line. (**b**) In situ hybridization of embryos after 10 days of culture on medium. (**b1**–**b4**) The wild-type embryo. (**b5**–**b8**) the OE49 embryo. The scales for b1 to b4 (and b5 to b8) are 205, 101, 50, and 25 µm, respectively. (**c**) Toluidine blue staining of embryos after 10 days of culture on medium. (**c1**,**c2**) The wild-type plumule. (**c3**,**c4**) The OE49 plumule. The scales for c1 and c2 (also c3 and c4) are 100 and 50 µm, respectively. PL: plumule; S: scutellum.

High homology was found between *ZmCYCB1-1* and *OsCYCB1-1* by the phylogenetic tree analysis, and previous studies have found that *OsCYCB1-1* could affect embryo development [22]. We therefore examined the effect of *ZmCYCB1-1* overexpression on embryo development in maize. After 7 days of in vitro culture, only OE49 embryos had green tissues (Figure 3(a2)). After 10 days of culture, both OE49 and WT embryos germinated, and the shoot length of OE49 were significantly greater than that of the WT (bud length was 6.2 mm and 3.3 mm for OE49 and the WT) (Figure 3(a3)). These results indicate that *ZmCYCB1-1* promotes the growth of isolated embryos.

We utilized in situ hybridization experiments on embryo sections to determine the localization of *ZmCYCB1-1*. The probe (310115-P: TTGGGAAGAAAAAAGAACAGACATCAGGGACCGC) was designed based on the *ZmCYCB1-1* genome sequence and used to hybridize with embryo sections. Consistent with the qRT-PCR results, signal was stronger in OE49 (Figure 3(b5–b8)) plants compared with WT (Figure 3(b1–b4)) under the 205, 101, 50, 25µm field of view, respectively. In both cases, *ZmCYCB1-1* expression was primarily detected in the nucleus of the plumule (PL) of the embryo. This finding suggests that *ZmCYCB1-1* is highly expressed in the nucleus of maize PL cells, where it positively regulates mitosis to accelerate the growth of isolated embryos.

The embryos were also sectioned and stained with toluidine blue, and OE49 cells showed stronger staining compared with the WT under the 100 µm field of view, suggesting that OE49 had more nuclei in the PL (Figure 3(c1,c3)), possibly due to accelerated mitosis. Zoom in to 50 µm to observe that the OE49-PL cells exhibited tighter junctions, and were more regular in shape, smaller and denser, with larger nuclei (Figure 3(c2,c4)). These findings indicate that perhaps the OE49-PL have higher mitotic activity than the WT-PL, which may underlie the higher growth rate of OE49 embryos. 

### 2.4. ZmCYCB1-1 Overexpression Increases Kernel Size 

**Figure 4:**
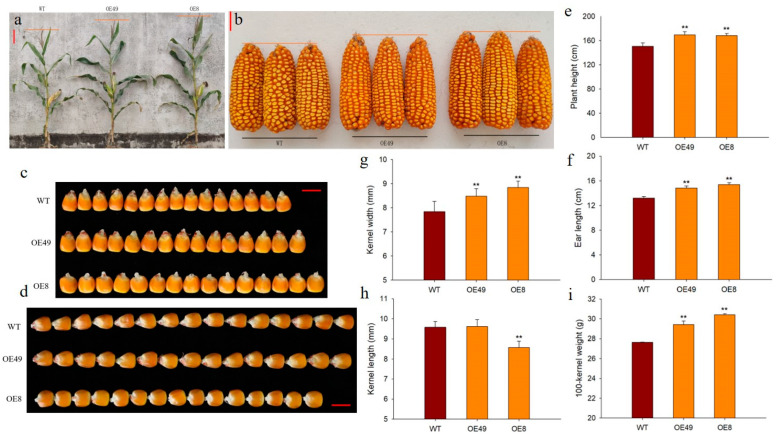
Phenotypic analysis of *ZmCYCB1-1* overexpression lines. (**a**,**e**) Plant height of the WT and *ZmCYCB1-1* overexpression plants. (**b**,**f**). Ear length of the WT and *ZmCYCB1-1* overexpression plants. (**c**,**g**) Kernel width of the WT and *ZmCYCB1-1* overexpression plants. (**d**,**h**) Kernel length of the WT and *ZmCYCB1-1* overexpression plants. (**i**) 100-kernel weight of the WT and *ZmCYCB1-1* overexpression plants. Scale bars are 10 cm in (**a**), 3 cm in (**b**), and 1cm in (**c**,**d**). Student’s *t* test, ** *p* < 0.01.

To explore the roles of *ZmCYCB1-1* in plant growth and development, we measured several phenotypic traits of OE49, OE8, and WT, including plant height, ear length, and 100-kernel weight. We found that the plant height of OE49 and OE8 increased by 12.38% and 11.85%, respectively, compared with the WT (Figure 4a,e). In addition, the ear length of OE49 and OE8 increased by 12.25% and 16.41% (Figure 4b,f), and 100-kernel weight by 6.34% and 9.97%, respectively (Figure 4i). The kernel width of OE49 and OE8 increased by 8.16% and 17.18%, respectively, compared with the WT (Figure 4c,g). OE49 showed no significant changes in kernel length, whereas the kernel length of OE8 decreased by 10.57% compared with the WT (Figure 4d,h). These results demonstrate that the increase in 100-kernel weight observed in OE49 and OE8 plants was largely due to changes in kernel width. Taken together, these findings reinforce the idea that *ZmCYCB1-1* positively impacts the vegetative and reproductive growth of maize, thereby increasing maize yield.

### 2.5. ZmCYCB1-1 Knockout Inhibits Plant Growth

To further investigate the function of *ZmCYCB1-1* in maize growth and development, we used CRISPR/Cas9 to generate *ZmCYCB1-1* knockout mutants in maize inbred line KN5585. Two homozygous knockout lines, KO1 and KO5, were obtained and confirmed by PCR amplification. KO1 had a 222-bp deletion between target sites 1 and 2, whereas KO5 carried a 222-bp inversion between target sites 1 and 2 (Figure S1a). Phenotypic examination indicated that the plant height (Figure S1c), stem diameter (Figure S1d), leaf length (Figure S1e), leaf width (Figure S1f), and leaf area (Figure S1g) of KO1 and KO5 at the V6 stage were reduced to varying degrees compared with the WT, indicating that the knockout of *ZmCYCB1-1* inhibits plant growth (Figure S1b). 

### 2.6. Transcriptome Analysis of ZmCYCB1-1 Overexpression and Knockout Plants

**Figure 5:**
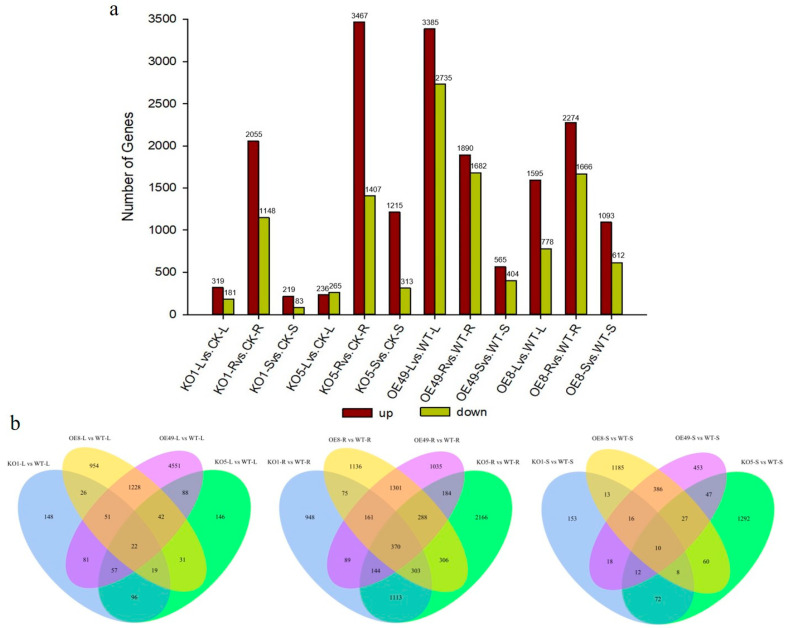
Statistical analysis of all DEGs identified in the leaves (L), stems (S), and roots (R) of KO1, KO5, OE49, and OE8. (**a**) Statistical analysis of the number of up-regulated (up) and down-regulated (down) DEGs. (**b**). Venn diagrams showing the distribution of DEGs.

To understand how *ZmCYCB1-1* overexpression and knockout affect gene expression patterns, we performed transcriptome sequencing on the leaves, roots, and stems of two overexpression lines (OE49 and OE8), two knockout lines (KO1 and KO5), and control plants (WT). When comparing the KO lines to WT, most DEGs were found in root tissues. KO1 and KO5 had 2055 and 3467 up-regulated genes and 1148 and 1407 down-regulated genes when compared to the WT, respectively. In OE lines, DEGs were mostly found in comparisons involving leaves, roots, and stems. For example, there were 3385 and 1595 up-regulated genes and 2735 and 778 down-regulated genes in the leaves of OE49 and OE8 compared with the WT, respectively. In addition, there were 1890 and 2274 up-regulated genes and 1682 and 1666 down-regulated genes in the roots of OE49 and OE8 compared with the WT, respectively. Finally, there were 565 and 1093 up-regulated genes and 404 and 612 down-regulated genes in the stem of OE49 and OE8 compared with the WT, respectively (Figure 5a).

**Figure 6:**
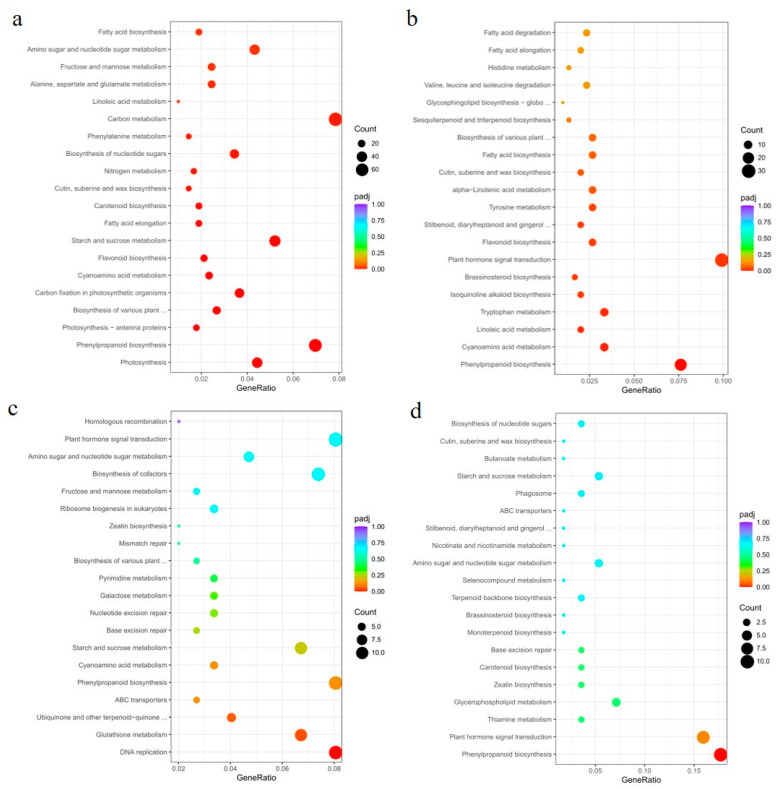
KEGG pathway enrichment analysis of DEGs between the OE and KO lines. (**a**) KEGG pathway analysis of DEGs identified between OE49 and WT leaves. (**b**) KEGG pathway analysis of DEGs identified between OE8 and WT leaves. (**c**) KEGG pathway analysis of DEGs identified in the roots of KO1 and KO5 compared with the WT. (**d**) KEGG pathway analysis of DEGs identified in the roots of KO1, KO5, OE49, and OE8 compared with the WT.

Next, we constructed Venn diagrams to examine the overlap of DEGs between different comparisons. A total of 4551 and 954 DEGs were found in the leaves of OE49 and OE8, respectively (Figure 5b). KEGG pathway enrichment analysis revealed that the OE49 DEGs were enriched in phenylpropanoid biosynthesis, starch and sucrose metabolism, and photosynthesis (Figure 6a), whereas the OE8 DEGs were enriched in plant hormone signal transduction and phenylpropanoid biosynthesis (Figure 6b). We also performed GO enrichment analysis of the DEGs, which revealed that the 40 DEGs between WT and OE49 leaves were enriched in photosynthesis (Figure S2a), and the 47 DEGs between WT and OE8 leaves were enriched in lipid metabolism (Figure S2b). In roots, 1113 DEGs were found in comparisons involving KO1 and KO5, and 370 DEGs were found in comparisons involving OE49, OE8, KO1, and KO5 (Figure 5b). KEGG enrichment analysis revealed the enrichment of these 1113 DEGs in terms associated with DNA replication (Figure 6c). All DEGs between the OE and KO lines were enriched in phenylpropanoid biosynthesis (Figure 6d). A total of 386 DEGs were found in comparisons of the WT stems to OE49 and OE8 stems (Figure 6b), and these 386 DEGs were enriched in MAPK signaling pathway-plant, phenylpropanoid biosynthesis, and plant hormone signal transduction (Figure S2c). 

To better understand the molecular mechanisms by which the overexpression of *ZmCYCB1-1* accelerates embryo growth rate in vitro, the embryos of OE49 and WT were used for transcriptome sequencing. After quantification and differential expression testing, a total of 3665 differentially expressed genes (DEGs) were obtained, of which 2272 were up-regulated and 1383 were down-regulated in OE49 embryos compared with the WT. We next performed GO enrichment analysis of the 3665 DEGs and found that they were significantly enriched in terms associated with photosynthesis, carbohydrate metabolic process, and oxidoreductase activity (Figure S3a). KEGG pathway analysis on DEGs with FDR < 0.05 indicates that they were enriched for photosynthesis-antenna protein, carbon fixation in photosynthetic organism, and photosynthesis (Figure S3b). In addition, pathway enrichment was performed by gene set enrichment analysis (GSEA) [25]. This analysis revealed that there were six gene sets with significant enrichment, including gene set 1 (AMPK signaling pathway), gene set 2 (carbon fixation in photosynthetic organisms), gene set 3 (cell cycle), gene set 4 (photosynthesis—antenna proteins), gene set 5 (photosynthesis), and gene set 6 (MAPK signaling pathway-plant). The GSEA results indicate that gene sets 1–5 were upregulated in OE49, whereas gene set 6 was downregulated in OE49 (Figure S4). To further verify the accuracy of our results, the relative expression levels of 15 genes selected randomly from the gene set 5 identified by GSEA were tested by qRT-PCR, genes and primers were listed in Supplemental Table S1. The results showed that the expression levels of 15 genes related to photosynthesis in OE49 embryos were increased to varying degrees compared with WT (Figure S5). These results demonstrated the reliability of the transcriptome analysis and further confirmed that overexpression of *ZmCYCB1-1* improved the photosynthesis in embryos. These results suggest that *ZmCYCB1-1* may regulate maize growth through alterations in phenylpropanoid biosynthesis, photosynthesis, and plant hormone signal transduction pathways. DNA replication was also affected by *ZmCYCB1-1* knockout. Notably, OE49 had a significant change in photosynthesis in leaves (Figure 6a), which is consistent with the results of transcriptome analysis of embryos (Figures S3 and S4). In OE8, the DEGs were enriched for plant hormone signal transduction pathways, including a number of auxin-responsive genes (Figure 6b). Finally, DEGs in OE49 and OE8 stems were enriched for MAPK signaling pathways, although the impact of such changes is not clear (Figure S2c).

## 3. Discussion

The cell cycle is the process by which eukaryotic cells replicate and divide; and it consists of two specific distinct phases: the interphase (including the G1; S; and G2 stages) and the mitosis phase (the M phase) [26]. Different cyclins are expressed at different stages of the cell cycle. In recent years, researchers have conducted in-depth studies on the periodic expression of cyclins in model plants, such as Arabidopsis [27,28,29], tobacco [16,19,30], and alfalfa [18,31,32]. Cyclin B1 has been shown to function at the G2/M transition and during the progression of M-phase [33]. Here, we used qRT-RCR to validate the gene expression profile at the vegetative and reproductive stages in *ZmCYCB1-1* overexpressing maize plants. *ZmCYCB1-1* expression was significantly up-regulated in many different tissues, such as young leaves at the VE stage; leaves, stems, and roots at the jointing stage; tassel and ears at the tasseling stage; and seeds at 10 DAP and 16 DAP.

A clustering analysis of CYCB genes in maize, rice, and Arabidopsis indicated that the maize *CYCB1-1* had the highest homology with rice *CYCB1-1*, and research has shown that in rice, *OsCYCB1-1* expression is critical for embryo and endosperm formation via the regulation of mitotic division [22]. We speculated that *ZmCYCB1-1* may have a similar function during seed development in maize. Indeed, in vitro culture of embryos revealed that overexpression of *CYCB1-1* significantly accelerated the induction of green tissue and the growth of embryos. Subsequent histological observation showed that cells in the plumule *ZmCYCB1-1* overexpression lines were smaller and more tightly packed, with larger nuclei in higher densities. These results are similar to earlier reports in the histological examination of maize embryogenic callus (EC) [34]. Our analysis of the transcriptomic data revealed the upregulation of photosynthesis-related genes in the embryos of *ZmCYCB1-1* overexpression lines compared with the WT. The regeneration ability and developmental rate of maize embryos are two key factors that restrict the efficiency of maize genetic transformation. The improved histological characteristics and increased growth rate exhibited by the embryos of *ZmCYCB1-1* overexpression plants could therefore be used to shorten the explant culture period. 

A higher expression of *SIKLUH*, which affects seed size, has been shown to increase cell proliferation in tomato near-isogenic lines [35], whereas a lower expression of *SIKLUH* can affect the expression of genes involved in lipid metabolism [36]. Recent studies have also shown that lipid metabolism regulates grain size and yield in rice (Nipponbare, ZH11 and HJ19) [37]. Our transcriptomic analysis revealed that the expression of several genes associated with this process was altered in *ZmCYCB1-1* overexpression lines compared with the WT. A total of 47 DEGs between OE8 to WT leaves were enriched in lipid metabolism pathways (Figure S2b), and among the 29 DEGs in the plant hormone signal transduction pathway, 13 encoded auxin receptor proteins, suggesting that phenotypic changes in OE8 relative to the WT were likely associated with auxin signal transduction and lipid metabolism. We also found 40 DEGs that were enriched in the photosynthesis pathways when comparing the leaves of OE49 and WT (Figure S2a). These DEGs may affect plant growth and development by modulating photosynthesis and the accumulation of starch and sucrose. However, elucidation of the mechanism by which *ZmCYCB1-1* affects photosynthesis and lipid metabolism requires additional study. In general, transcriptome analysis revealed that overexpression of *ZmCYCB1-1* can regulate photosynthesis and lipid metabolism pathways, and affect carbohydrate metabolism during plant growth. The final performance is a positive regulation of plant growth and seed development. Conversely, when *ZmCYCB1-1* was deleted, transcriptome analysis reveals changes in the expression of genes related to DNA replication, the normal cell cycle activity might be affected, and the plants exhibited reduced growth rate; therefore, demonstrating further proof that *ZmCYCB1-1* plays an important role in regulating the normal growth and development of plants.

Seed weight is a key determinant of yield; however, seed development is a complex process that requires coordinated integration of genetic, metabolic, physiological, and environmental factors [38]. In plants, seed size and weight depend primarily on cell number and cell size. Published data have shown that cell number generally makes a larger contribution to seed size than cell size [39]. A growing number of studies have shown that cell cycle regulators and differences in cell cycle type play important roles in the growth, development, and function of seeds [40,41,42]. For example, the overexpression of *KRP1* and *KRP2*, two cyclin-dependent kinase inhibitors, resulted in a significant reduction in grain filling and seed germination in rice [41,43]. In Arabidopsis, downregulation of the CDK inhibitor *ICK/KRP* upregulates the E2F pathway and increases cell proliferation and organ and seed size [42,44]. The Arabidopsis *CYCB1-4* overexpression lines exhibited normal development but increased seed size due to accelerated cell cycle progression [23]. In our study, the overexpression of *ZmCYCB1-1* resulted in smaller embryo cells with a higher density and increased the number of cells per unit area, which likely contributed to an increased seed size. Maize seed development requires the coordination of embryo and endosperm, in vitro culture of embryos experiment indicated that *ZmCYCB1-1* gene played a very positive role in promoting the development of embryos; however, whether overexpression of *ZmCYCB1-1* affects the development of endosperm still needs further study. Furthermore, the overexpression plants were taller and produced longer ears compared with the WT. Specifically, the increase in kernel weight of OE49 was primarily due to an increased kernel width. Although OE8 exhibited a decreased kernel length, the increase in kernel width compensated for this loss, leading to a net gain in kernel weight. On the whole, in our study, the overexpression of *ZmCYCB1-1* in maize resulted in an increased ear length and an enhanced kernel weight by increasing kernel width. Considering the effects of genotype and environmental factor of crop yield, it is necessary to further carry out multi-year, multi-environment evaluation and large-scale field test in order to appraise the potential value of *ZmCYCB1-1* gene in breeding. Additionally, the wild-type plants can be crossed with overexpressed and knockout plants, respectively, to further clarify the genetic effect of *ZmCYCB1-1* and inform breeding efforts of maize. This study represents the first systematic examination of the maize *CYCB1-1* gene, which was found to significantly impact plant growth and kernel size, with implications for future yield improvement efforts. 

## 4. Materials and Methods

### 4.1. Plant Materials and Growth Conditions

The maize inbred line ZPM1 was used for gene cloning and was provided by the Maize Research Institute of Beijing Academy of Agriculture and Forestry Sciences (BAAFS) (Beijing, China). The inbred line KN5585 was utilized for transformation. All T0 overexpression and edited plants were grown in the greenhouse of BAAFS. The T1 to T3 generation of overexpression lines were grown in a field at the Agricultural Experiment Station of BAAFS (Beijing, China).

### 4.2. Cloning and Clustering Analysis of the ZmCYCB1-1 Gene

The reference sequence of *ZmCYCB1-1* was obtained from the gene database of the National Center for Biotechnology Information (NCBI). Genomic DNA was extracted from young leaves of the maize inbred line ZPM1 using a plant DNA extraction kit (TIANGEN, Beijing, China). Primers used to amplify the full-length genomic sequence of *ZmCYCB1-1* were designed using PRIMER5 software [45] (310115F: 5′-ATGCCCACGCGCAACCACAA-3′; 310115R: 5′-TTACTTGATCTCCACTGCGG-3′). The full-length genomic DNA sequence of *ZmCYCB1-1* was amplified using high-fidelity DNA polymerase KOD-Plus-Neo (Toyobo Life Science, Osaka, Japan). 

For B-type cyclin cluster analysis, an unrooted neighbor-joining phylogenetic tree of nine genes in Arabidopsis, five genes in rice, and seven genes in maize was generated by MEGA7.0 using full-length protein sequences [46]. Including *AtCYCB1-2* (AT5G06150), *AtCYCB1-4* (AT2G26760), *AtCYCB3-1* (AT1G16330), *AtCYCB2-4* (AT1G76310), *AtCYCB1-5* (AT1G34460), *AtCYCB2-1* (AT2G17620), *AtCYCB1-1* (AT4G37490), *AtCYCB1-3* (AT3G11520), *AtCYCB2-3* (AT1G20610), *OsCYCB1-1* (LOC_Os01g59120); *OsCYCB1-3* (LOC_Os01g17402), *OsCYCB2-2* (LOC_Os06g51110), *OsCYCB1-5* (LOC_Os05g41390), *OsCYCB2-1* (LOC_Os04g47580), *ZmCYCB1-1* (Zm00001d012560); *ZmCYCB1-2* (Zm00001d010656), *ZmCYCB1-3* (Zm00001d008221), *ZmCYCB1-4* (Zm00001d049105), *ZmCYCB1-5* (Zm00001d043164), *ZmCYCB2-1* (Zm00001d036360) and *ZmCYCB2-2* (Zm00001d002662) (Figure 1 and Table 1). 

### 4.3. Vector Construction and Genetic Transformation

To construct an overexpression vector, the 2323 bp genomic sequence of *ZmCYCB1-1* was amplified by PCR using primers 310115F and 310115R and inserted downstream of the ubiquitin promoter into the pCUbi3301 vector to generate the pUbi::ZmCYCB1-1 construct [47]. To disrupt the *ZmCYCB1-1* gene, two guide RNAs were designed using a CRISPR design tool (http://crispr.hzau.edu.cn/CRISPR2/, accessed on 20 May 2022) (Target1: TCAGCTCGTGAAGAACGTGCAGG, Target2: ACTATGACATCCTCAGGAGGCGG) to target the 3rd and 4th exon [48,49]. The immature embryos of the maize inbred line KN5585 were used for *Agrobacterium tumefaciens* (strain EHA105)-mediated transformation of all vector types [50].

### 4.4. In Vitro Growth of Immature Embryos of ZmCYCB1-1 Overexpression Plants

The ears of OE49 and WT were sampled at the 10 DAP, sterilized in 10% sodium hypochlorite for 10 min, and rinsed three times in sterile deionized water. Embryos were collected and inoculated in MS medium and grown under sterile conditions at 16 h light/8 h dark cycles at 25 °C. After 7 and 10 days of inoculation, the embryos were phenotyped for bud growth, including rate and length.

### 4.5. Expression Analysis of ZmCYCB1-1 

WT plants and transgenic maize plants that overexpressed *ZmCYCB1-1* were grown under identical greenhouse conditions. For expression analysis, samples were harvested from *ZmCYCB1-1* overexpression plants at different developmental stages. Leaves and roots were collected at the VE (when the first true leaf emerged from the coleoptile, BBCH 10), V1 (when the first leaf fully expanded, BBCH 11), and V2 stage (when the second leaf fully expanded, BBCH 12). Leaves, stems, and roots were collected at the V6 stage (when the sixth leaf fully expanded and the plants were at the jointing stage, BBCH 16-30). The tassel and ear samples were collected at the VT stage (the tasseling stage, BBCH 51-61), and seeds were collected at 10 and 16 DAP (BBCH 71-79) [51]. All samples were snap-frozen in liquid nitrogen stored at −80 °C. Total RNA was extracted using a Total RNA Extraction Kit (TIANGEN, China), following the manufacturer’s protocol. One microgram of RNA was then used as the template for reverse transcription with a HiFi Script gDNA Removal RT Master Mix (CWBIO, Beijing, China). Appropriate dilutions of the cDNA were used in the qRT-PCR reaction, using Power Up™ SYBR™ Green Master Mix (ABI) (Thermo Fisher Scientific, Waltham, MA, USA). Two primers (CYCB-F: 5′TAGTGTGGCCATCCAAGCAT-3′; SYBR-R: 5′ ATGACCTTCTTCCTCGAGCG-3′) designed based on the sequence of *ZmCYCB1-1* were used for qRT-PCR. The amplification of glyceraldehyde-3-phosphate deHaseN (*gpn1*) (gpn-F: 5′-TGACCAAGGTGAAGAGCACTGT-3′; gpn-R: 5′-CAAATCTCACGTGGCTATGAAAC-3′) were used as an internal control. Experiments were independently replicated three times under identical conditions. The relative expression levels of the target genes were calculated using the 2^−^^ΔΔCt^ method [52]. Student’s *t*-test was used for significance analysis with significance level (alpha) = 0.05.

### 4.6. In Situ Hybridization and Toluidine Blue Staining

After growing for 10 days on MS medium, the embryos were fixed with formalin-acetic acid-alcohol (FAA) fixative. Paraffin-embedded tissue sections were dewaxed in xylene and rehydrated through graded alcohol–water mixtures. Proteinase K (20ug/mL) was then added, followed by incubation at 37 °C for 20 min. Endogenous peroxidase was blocked with 3% H_2_O_2_ in methanol for 10 min. Probes (linc00173/linc00173 TSV2) were then added to the mixture, followed by incubation in a humidity chamber to hybridize overnight at 42 °C. Samples were then washed for 10 min once with 2× SSC, followed by two 10-min washes with 1× SSC at 37 °C. After washing, blocking solution was added (normal rabbit serum), followed by incubation with mouse anti-digoxigenin antibody overnight at 4 °C. Color was developed using DAB and counterstaining with hematoxylin. Dehydration was performed through an alcohol gradient of 75%, 85%, 100%, and n-butanol, for 6 min at each step. Samples were then visualized using a Nikon Eclipse Ci microscope (Nikon, Minato City, Tokyo, Japan).

Sections were placed into ethylene glycol ethyl ether acetate, incubated for 6 h at 37 °C, overnight at 37 °C, and for 10–15 min at room temperature twice. The sections were rehydrated in 100%, 95%, 90%, and 80% alcohol, for 10 min at each step. Finally, samples were rinsed with running water. The sections were placed into a toluidine blue staining solution for 2 min, then washed and observed under a Nikon Eclipse E100 microscope (Nikon, Minato City, Tokyo, Japan).

### 4.7. RNA-Seq Analysis

Total RNA was extracted from the samples using the RNeasy Plant Mini Kit (TIANGEN, Beijing, China), following the manufacturer’s instructions. A Nanodrop 2000 spectrophotometer (Thermo Fisher Scientific) was used to determine the concentration and evaluate the purity of RNA samples. An Agilent 2100 Bioanalyzer and 2100 RNA nano 6000 assay kit (Agilent Technologies, Santa Clara, CA, USA) were used to evaluate the integrity of the RNA samples. After QC, the poly-A RNA was enriched by a TIAN Seq mRNA Capture Kit (TIANGEN, Beijing, China). Then, using the captured RNA as the starting sample, a TIAN Seq Fast RNA Library Kit (TIANGEN, Beijing, China) was used to construct the transcriptome sequencing libraries. Library concentration was first quantified using a Qubit 2.0 fluorometer (Thermo Fisher Scientific, Waltham, MA, USA), and then diluted to 1 ng/µL before checking insert size on an Agilent 2100 and quantifying to greater accuracy via qRT-PCR (library activity > 2 nM). Clustering of the index-coded samples was performed on a cBot Cluster Generation System using TruSeq PE Cluster Kit v3-cBot-HS (Illumina, San Diego, CA, USA), according to the manufacturer’s instructions. After cluster generation, the libraries were sequenced on an Illumina sequencing platform and 150 bp paired-end reads were generated. The RNA-Seq reads were mapped to the maize reference genome Zm-B73-REFERENCE-NAM-5.0 using TopHat [53]. KOBAS software was used to analyze the statistical enrichment of DEGs in KEGG (Kyoto Encyclopedia of Genes and Genomes) pathways [54]. GO enrichment analysis of DEGs was performed by ShinyGO (http://bioinformatics.sdstate.edu/go/, accessed on 20 May 2022).

## 5. Conclusions

In this study, we investigated the roles of the maize *ZmCYCB1-1* gene in the regulation of embryo and seed development in maize. Overexpression of *ZmCYCB1-1* resulted in accelerated embryo development, increased kernel size, and increased kernel weight. These results provide new insights into the regulatory functions of cyclins during seed development and represent an important step toward utilizing cyclins to enhance maize yield.

## Figures and Tables

**Table 1:** Basic characteristics of CYCB family genes in Arabidopsis, rice, and maize.

Gene	Locus	Chromosome	Location	Number of AA	MW (kDa)	PI
AtCYCB1-2	AT5G06150	5	1859279..1861703	445	49.8	9.32
AtCYCB1-4	AT2G26760	2	11401118..11403433	387	43.6	7.12
AtCYCB3-1	AT1G16330	1	5582387..5587461	648	72.4	9.91
AtCYCB2-4	AT1G76310	1	28627694..28630789	431	49.2	5.65
AtCYCB1-5	AT1G34460	1	12595110..12602379	491	55.4	9.14
AtCYCB2-1	AT2G17620	2	7664055..7666522	429	49.2	5.38
AtCYCB1-1	AT4G37490	4	17621886..17624308	428	48.5	8.47
AtCYCB1-3	AT3G11520	3	3625286..3627273	414	46.3	9.02
AtCYCB2-3	AT1G20610	1	7134659..7137828	429	48.9	5.44
OsCYCB1-1	LOC_Os01g59120	1	34149456..34152432	448	49.5	9.12
OsCYCB1-3	LOC_Os01g17402	1	10011887..10015563	423	47.4	9.37
OsCYCB2-2	LOC_Os06g51110	6	30915116..30918901	419	47.5	5.69
OsCYCB1-5	LOC_Os05g41390	5	24240406..24243654	449	49.3	9.48
OsCYCB2-1	LOC_Os04g47580	4	28218757..28221766	418	47.1	5.31
ZmCYCB1-1	Zm00001d012560	8	177903858..177906686	449	50	9.19
ZmCYCB1-2	Zm00001d010656	8	122821167..122824013	446	48.3	9.49
ZmCYCB1-3	Zm00001d008221	8	1820085..1823293	479	53.3	9.22
ZmCYCB1-4	Zm00001d049105	4	16351535..16352295	192	22.2	5.07
ZmCYCB1-5	Zm00001d043164	3	192558950..192561719	442	49.5	9.14
ZmCYCB2-1	Zm00001d036360	6	92281211..92285039	424	47.7	5.75
ZmCYCB2-2	Zm00001d002662	2	18900412..18903957	424	47.5	5.5

AA: amino acids; MW: molecular weight; PI: isoelectric point of the deduced polypeptide.

## Data Availability

All data generated or analyzed during this study are included in this published article.

## Supplementary Materials

The following supporting information can be downloaded at: https://www.mdpi.com/article/10.3390/ijms23115907/s1.
