# Supplementary material for: The B-Type Cyclin CYCB1-1 Regulates Embryonic Development and Seed Size in Maize
Source: Int J Mol Sci. 2022 May 25;23(11):5907. doi: 10.3390/ijms23115907 (PMC9180882; doi:10.3390/ijms23115907)
Supplement: Supplementary file 1 [file ijms-23-05907-s001.zip › ijms-1726783 - Authorship Change Form.pdf]

Multidisciplinary
Digital Publishing
Institute

## *International Journal of Molecular Sciences* — Change of Authorship Form

(Must be Completed and Signed by ALL Authors)

Manuscript ID: ijms-1726783
Manuscript Title: The B-type cyclin CYCB1-1 regulates embryonic development and seed size in maize

Description of the change: new author have been added

**Original Authorship**

LIST ALL AUTHORS in the same order as the original (first) submission. For more than 10 use an extra sheet.

| | Name | Affiliation |
|---|---|---|
| author (1) | Bingbing Zhao | College of Agronomy, Jiangxi Agricultural University; Maize Research Institute, Beijing Academy of Agriculture & Forestry Sciences/Beijing Key Laboratory of Maize DNA Fingerprinting and Molecular Breeding |
| author (2) | Miaoyi Zhou | Maize Research Institute, Beijing Academy of Agriculture & Forestry Sciences/Beijing Key Laboratory of Maize DNA Fingerprinting and Molecular Breeding |
| author (3) | Wen Ren | Maize Research Institute, Beijing Academy of Agriculture & Forestry Sciences/Beijing Key Laboratory of Maize DNA Fingerprinting and Molecular Breeding |
| author (4) | Hanshu ai Li | Maize Research Institute, Beijing Academy of Agriculture & Forestry Sciences/Beijing Key Laboratory of Maize DNA Fingerprinting and Molecular Breeding |
| author (5) | Qian Zhang | College of Life Science, Yangtze University |
| author (6) | Ya Liu | Maize Research Institute, Beijing Academy of Agriculture & Forestry Sciences/Beijing Key Laboratory of Maize DNA Fingerprinting and Molecular Breeding |
| author (7) | Haohua He | College of Agronomy, Jiangxi Agricultural University |
| author (8) | | |
| author (9) | | |
| author (10) | | |

**New Authorship**

All authors must sign below agreeing to the new changes in authorship. The authorship order must match the new title page of the manuscript. Signatures below certify compliance with the author responsibilities on the next page. List ALL AUTHORS in the same order as the new version.

| | Title (Mr./Ms./Mrs./Dr./Prof.) | Name | Email | Affiliation | Signature& Date |
|---|---|---|---|---|---|
| author (1) | Dr. | Bingbing Zhao | bing3015@126.com | College of Agronomy, Jiangxi Agricultural University; Maize Research Institute, Beijing Academy of Agriculture & Forestry Sciences/Beijing Key Laboratory of Maize DNA Fingerprinting and Molecular Breeding | Bing bing Zhao 2022.5.20 |
| author (2) | Ms. | Miaoyi Zhou | monazhou001@126.com | Maize Research Institute, Beijing Academy of Agriculture & Forestry Sciences/Beijing Key Laboratory of Maize DNA Fingerprinting and Molecular Breeding | Miaoyi Zhou 2022.5.20 |
| author (3) | Dr. | Wen Ren | renwen@maizedna.org | Maize Research Institute, Beijing Academy of Agriculture & Forestry Sciences/Beijing Key Laboratory of Maize DNA Fingerprinting and Molecular Breeding | Wen Ren. 2022.5.20 |
| author (4) | Mr. | Hanshuai Li | lhswgy@126.com | Maize Research Institute, Beijing Academy of Agriculture & Forestry Sciences/Beijing Key Laboratory of Maize DNA Fingerprinting and Molecular Breeding | Hanshuai Li 2022.5.20 |
| author (5) | Ms. | Qian Zhang | zg0502q@163.com | College of Life Science, Yangtze University | Qian Zhang 2022.5.20 |
| author (6) | Prof. Dr. | Guangming He | heguangming@pku.edu.cn | School of Life Sciences and School of Advanced Agriculture Sciences, Peking-Tsinghua Center for Life Sciences, Peking University | Guangming He 2022.5.20 |
| author (7) | Prof. Dr. | Ya Liu | liuya@maizedna.org | Maize Research Institute, Beijing Academy of Agriculture & Forestry Sciences/Beijing Key Laboratory of Maize DNA Fingerprinting and Molecular Breeding | Ya Liu 2022.5.20 |
| author (8) | Prof. Dr. | Haohua He | hhhua64@163.com | College of Agronomy, Jiangxi Agricultural University | Haohua He 2022.5.20 |

**Authors to be removed (If any)**

| | name | affiliation | Signature&Date |
|---|---|---|---|
| author (1) | | | |
| author (2) | | | |
| author (3) | | | |
| author (4) | | | |
| author (5) | | | |

Please list all the author's Contribution here:

| Author's Contribution |
|---|
| author (1) Methodology; Software; Visualization; Writing – original draft; Writing – review & editing |
| author (2) Formal analysis; Software; Validation |
| author (3) Formal analysis; Validation; Visualization |
| author (4) Data curation; Investigation |
| author (5) Data curation; Investigation |
| author (6) Conceptualization; Formal analysis |
| author (7) Conceptualization; Funding acquisition; Project administration; Supervision; Writing – review & editing |
| author (8) Conceptualization; Project administration; Supervision; Writing – review & editing |
| author (9) |
| author (10) |

Contributor Roles Taxonomy (CRediT)

| | |
|---|---|
| Conceptualization | Ideas; formulation or evolution of overarching research goals and aims. |
| Data curation | Management activities to annotate (produce metadata), scrub data and maintain research data (including software code, where it is necessary for interpreting the data itself) for initial use and later re-use. |
| Formal analysis | Application of statistical, mathematical, computational, or other formal techniques to analyze or synthesize study data. |
| Funding acquisition | Acquisition of the financial support for the project leading to this publication. |
| Investigation | Conducting a research and investigation process, specifically performing the experiments, or data/evidence collection. |
| Methodology | Development or design of methodology; creation of models. |
| Project administration | Management and coordination responsibility for the research activity planning and execution. |
| Resources | Provision of study materials, reagents, materials, patients, laboratory |

| | |
|---|---|
| | samples, animals, instrumentation, computing resources, or other analysis tools. |
| Software | Programming, software development; designing computer programs; implementation of the computer code and supporting algorithms; testing of existing code components. |
| Supervision | Oversight and leadership responsibility for the research activity planning and execution, including mentorship external to the core team. |
| Validation | Verification, whether as a part of the activity or separate, of the overall replication/reproducibility of results/experiments and other research outputs. |
| Visualization | Preparation, creation and/or presentation of the published work, specifically visualization/data presentation. |
| Writing - original draft | Preparation, creation and/or presentation of the published work, specifically writing the initial draft (including substantive translation). |
| Writing - review & editing | Preparation, creation and/or presentation of the published work by those from the original research group, specifically critical review, commentary or revision – including pre- or post-publication stages. |
