# Supplementary material for: The B-Type Cyclin CYCB1-1 Regulates Embryonic Development and Seed Size in Maize
Source: Int J Mol Sci. 2022 May 25;23(11):5907. doi: 10.3390/ijms23115907 (PMC9180882; doi:10.3390/ijms23115907)
Supplement: Supplementary file 1 [file ijms-23-05907-s001.zip › ijms-1726783-supplementary after proofreading.pdf]

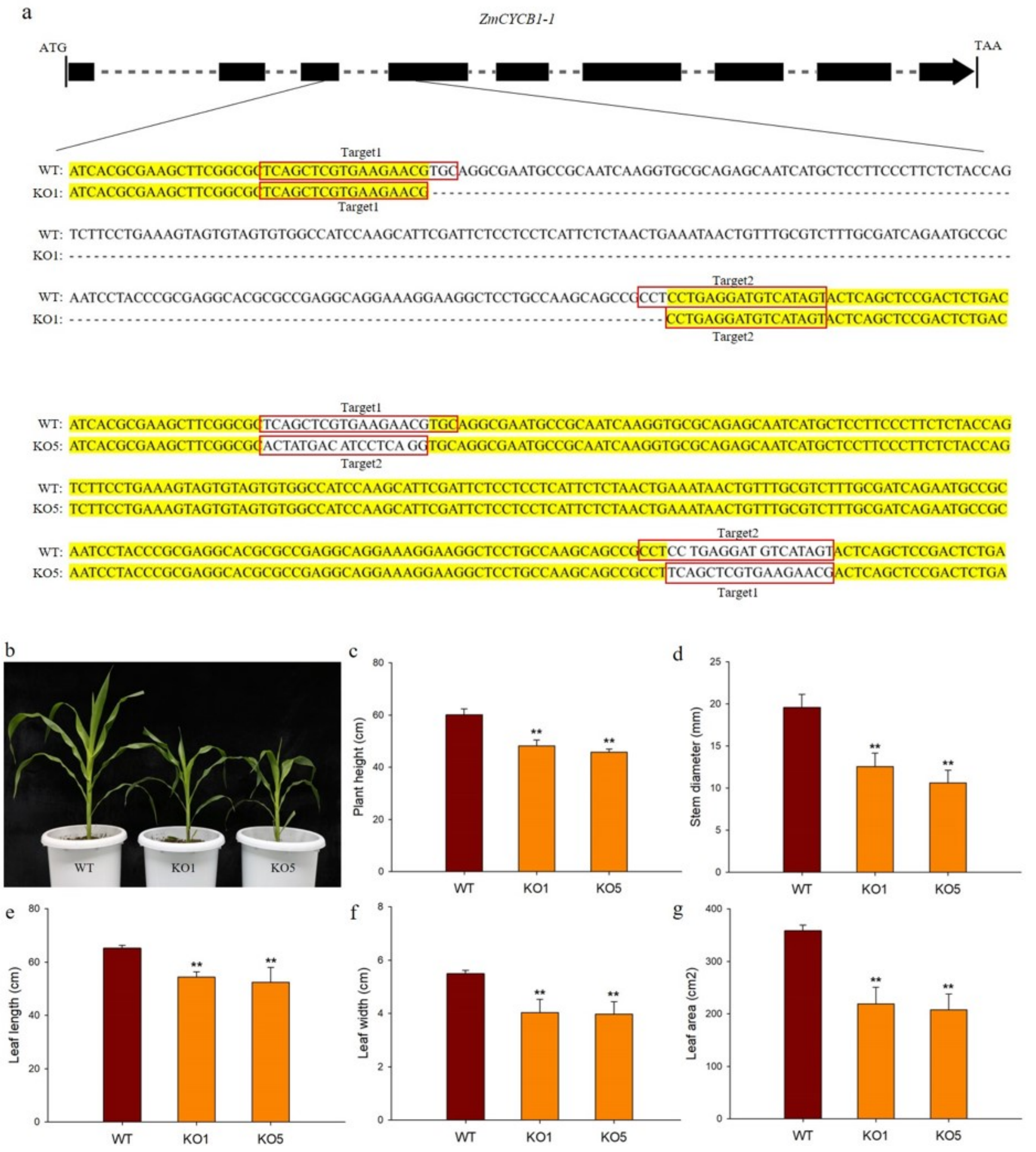

Figure S1. Phenotypic analysis of *ZmCYCB1-1* knockout lines. **a.** Alignment of Z*mCYCB1-1* sequences of the WT and two homozygous knockout lines (KO1 and KO5). Filled black squares represent exons, horizontal dotted lines represent introns, and the target sites are highlighted by red boxes, identical sequences are colored in yellow. **b.** the WT and *ZmCYCB1-1* knockout plants at the V6 stage. **c.** Plant height of the WT and *ZmCYCB1-1* knockout plants. **d.** Stem diameter of the WT and *ZmCYCB1-1* knockout plants. **e.** Leaf length of the WT and *ZmCYCB1-1* knockout plants. **f.** Leaf width of the WT and *ZmCYCB1-1* knockout plants. **g.** Leaf area of the WT and *ZmCYCB1-1* knockout plants. Student's *t* test, ** $p < 0.01$.

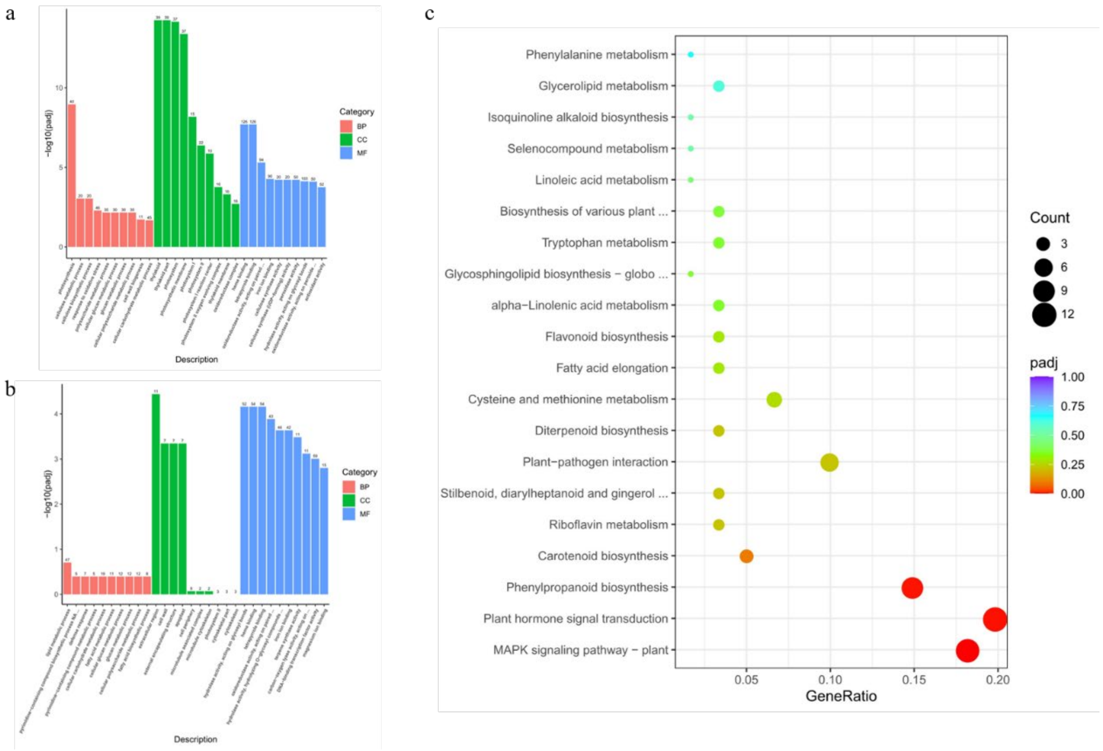

Figure S2. Functional enrichment analysis of DEGs in the OE lines. **a.** GO enrichment analysis of DEGs identified in OE49 leaves. **b.** GO enrichment analysis of DEGs identified in OE8 leaves. **c.** KEGG pathway analysis of DEGs identified in the stems of OE49 and OE8.

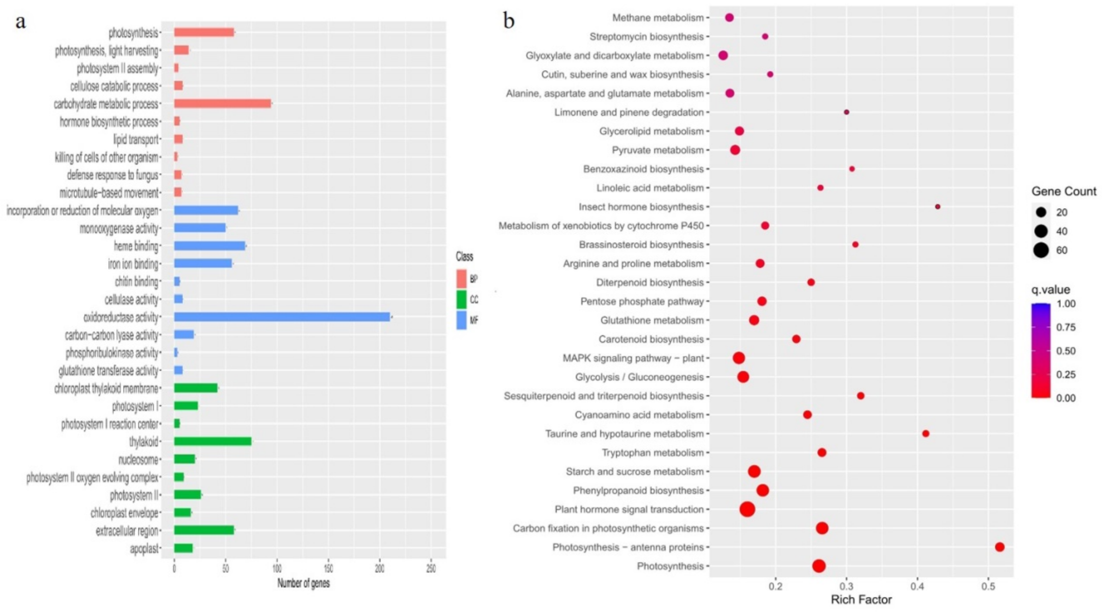

Figure S3. Functional enrichment analysis of DEGs between OE49 and WT embryos. **a.** GO Functional enrichment analysis of DEGs. **b.** KEGG pathway enrichment analysis of DEGs.

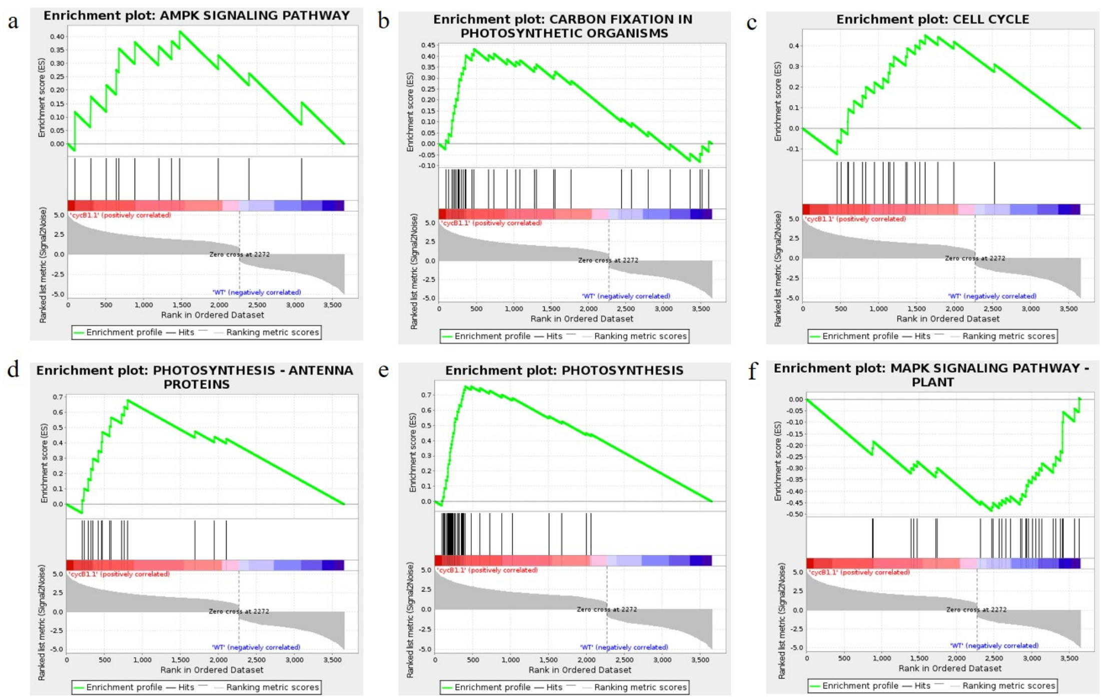

Figure S4. Gene set enrichment analysis (GSEA) of DEGs between OE49 and WT embryos. a-f represent gene sets 1-6.

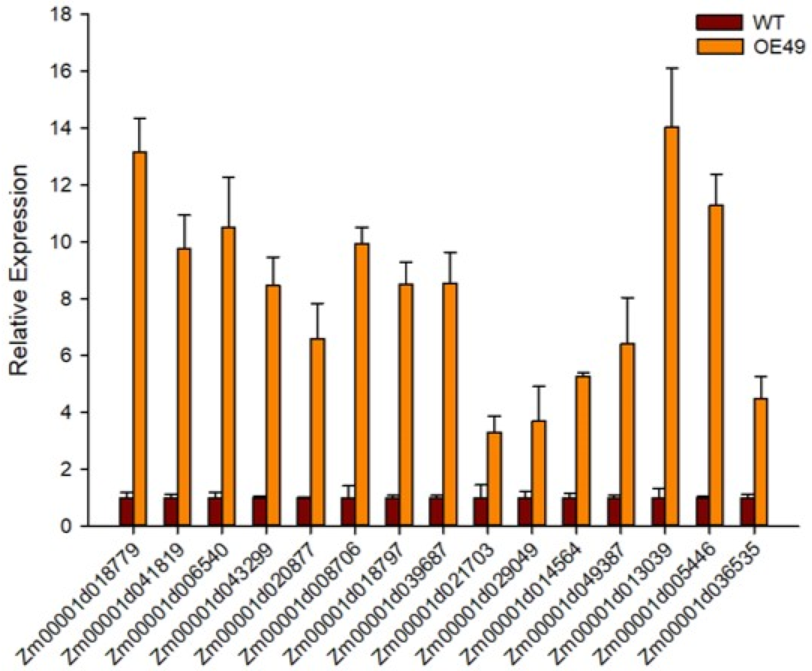

Figure S5. Relative expression levels of photosynthesis-related genes identified by GSEA in WT and OE49 embryos. The expression level of all genes in WT set to 1.

Table S1. Gene information related to photosynthesis in GSEA analysis.

Gene id	Description	Primers	Up-regulated
Zm00001d018779	oxygen-evolving enhancer protein 2, chloroplastic	F: 5′- CAAGCAGTACTACGCCGTGTCG-3′;<br>R: 5′- CAAGCAGTACTACGCCGTGTCG-3′	13.18-fold
Zm00001d041819	photosystem I reaction center subunit N, chloroplastic	F: 5′- CTTCTCATCCTGTGCCGTGT-3′;<br>R: 5′- GCTGCTAGCTGCTTCACCACT-3′	9.77-fold
Zm00001d006540	oxygen-evolving enhancer protein 3, chloroplastic	F: 5′- GCAAGCTCTTCAGCACCAT-3′;<br>R: 5′- CCATCTAGGCTAGCTTGGC-3′	10.51-fold
Zm00001d043299	oxygen-evolving enhancer protein 3, chloroplastic	F: 5′- TCCAGCAAAAGAGATCGAGA-3′;<br>R: 5′- ATATGCGAGCGTGAAAGTGA-3′	8.49-fold
Zm00001d020877	photosystem I reaction center subunit V, chloroplastic	F: 5′- CCACCTCAAGCAACGGATAC-3′;<br>R: 5′- AAATCCATGTACACGCGCAG-3′	6.6-fold
Zm00001d008706	photosystem I reaction center subunit XI, chloroplastic	F: 5′- GTGCTCTGGGCCTACTTTCT-3′;<br>R: 5′- CAATCCATCCATGTGCAAAG-3′	9.95-fold
Zm00001d018797	photosystem I reaction center subunit psaK, chloroplastic	F: 5′- TCAGACTCAGCAGCCTCGAT-3′;<br>R: 5′- AACAGGAGCTATATGTCCATTGG-3′	8.52-fold
Zm00001d039687	photosystem I reaction center subunit XI, chloroplastic	F: 5′- TTTCAGCAGTGACGAGCATC-3′;<br>R: 5′- ATGGCATTTCACTAGCTGGTT-3′	8.55-fold
Zm00001d021703	oxygen-evolving enhancer protein 3, chloroplastic	F: 5′- TGAGATCTGGGAACAGTGCA-3′;<br>R: 5′- CATTATTAGCCCTTGCGTCT-3′	3.3-fold
Zm00001d029049	photosystem II repair protein PSB27-H1, chloroplastic	F: 5′- CGAGGAATACGTGAGCGAGA-3′;<br>R: 5′- AGCTGATGTAATGGCCCGAC-3′	3.69-fold
Zm00001d014564	oxygen-evolving enhancer protein 1, chloroplastic	F: 5′- TGAGCGTCACCAAGAGCAAC-3′;<br>R: 5′- AAGTTCTCCATGACGGTCCG-3′	5.27-fold
Zm00001d049387	photosystem II 10 kDa polypeptide, chloroplastic	F: 5′- GCTGCGTACGTGATAGATCG-3′;<br>R: 5′- GCTGATGGGCAAGCAATAAG-3′	6.4-fold

Zm00001d013039	photosystem I reaction center subunit II, chloroplastic	F: 5′- CCCATCGACGTCAAGTTCA-3′;<br>R: 5′- CAAATTACACATCCATGTCCAC-3′	14.05-fold
Zm00001d005446	photosystem I reaction center subunit IV B, chloroplastic	F: 5′- CTACGCCTTGGACGAGATC -3′;<br>R: 5′- TTGAGTCCACGGTGAATGAA-3′	11.3-fold
Zm00001d036535	oxygen-evolving enhancer protein 1, chloroplastic	F: 5′- GGTAAGCACGCATGCATTGT-3′;<br>R: 5′- TCACCTCCATGTACGTTTTGC-3′	4.49-fold
